# Tolerance of clinical vancomycin-resistant *Enterococcus faecium* isolates against UV-C light from a mobile source

**DOI:** 10.1186/s13756-023-01259-3

**Published:** 2023-07-04

**Authors:** B. Knobling, G. Franke, C. Belmar Campos, H. Büttner, M. Christner, E. M. Klupp, P. M. Maurer, J. K. Knobloch

**Affiliations:** 1 Institute for Medical Microbiology, Virology and Hygiene, Department Infection Prevention and Control, University Medical Center Hamburg-Eppendorf, Hamburg, Germany; 2 Institute for Medical Microbiology, Virology and Hygiene, University Medical Center Hamburg-Eppendorf, Hamburg, Germany

**Keywords:** Automatic room decontamination, Disinfection, UV-C, UV-irradiation, vancomycin resistant enterococci

## Abstract

**Background:**

Admission to a room previously occupied by patients carrying environmentally robust pathogens implies an increased risk of acquiring those pathogens. Therefore, ‘No-touch’ automated room disinfection systems, including devices based on UV-C irradiation, are discussed to improve terminal cleaning. It is still unclear if clinical isolates of relevant pathogens behave differently under UV-C irradiation compared to laboratory strains used in the approval process of disinfection procedures. In this study we analysed the susceptibility of well characterized clonally divergent vancomycin-resistant enterococci (VRE) strains, including a linezolid-resistant isolate, against UV-C radiation.

**Methods:**

Susceptibility against UV-C of ten clonally divergent clinical isolates of VRE was determined in comparison to the commonly used test organism *Enterococcus hirae* ATCC 10541. Ceramic tiles contaminated with 10^5^ to 10^6^ colony forming units/25 cm² of the different enterococci were positioned at a distance of 1.0 and 1.5 m and irradiated for 20 s, resulting in a UV-C dose of 50 and 22 mJ/cm², respectively. Reduction factors were calculated after quantitative culture of the bacteria recovered from treated and untreated surfaces.

**Results:**

Susceptibility to UV-C varied considerably among the strains studied, with the mean value of the most robust strain being up to a power of ten lower compared to the most sensitive strain at both UV-C doses. The two most tolerant strains belonged to MLST sequence types ST80 and ST1283. The susceptibility of the laboratory strain *E. hirae* ATCC 10541 ranged between the most sensitive and most tolerant isolates for both irradiation doses. However, for UV-C dose of 22 mJ/cm², the reduction of the most tolerant isolate of ST1283 was statistically significantly lower compared to *E. hirae* ATCC 10541. The most susceptible strains belonged to the MLST sequence types ST117 and ST203.

**Conclusions:**

These results indicate that UV-C doses reported in the literature are sufficient for the reduction of commonly used reference strains of enterococci but could be insufficient for the reduction of tolerant patient VRE-isolates in a hospital setting. Therefore, for future studies, the most tolerant clinical isolates should be used to validate automated UV-C devices or longer exposure times should be expected to ensure efficacy in the real world.

**Supplementary Information:**

The online version contains supplementary material available at 10.1186/s13756-023-01259-3.

## Background

In recent decades, contamination of surfaces in the patient environment as cause for acquisition of nosocomial infections and transmission of pathogens has gained importance. Especially for *Staphylococcus aureus*, *Enterococcus faecium* and *Clostridioides difficile* survival on inanimate surfaces for days to months is well known [1, 2]. This potential of persistence results in an increased risk of acquiring such nosocomial pathogen if a patient is accommodated in a room, which previously was occupied by an infected or colonized patient [3]. In addition, contaminated inanimate surfaces become relevant for the transmission of pathogens due to failures in the process of surface disinfection and insufficient compliance with hand hygiene [4, 5].

Therefore, automatic room disinfection devices were discussed in the last decade as a possible solution to improve surface disinfection. Due to the safety and operational difficulties in using gaseous procedures or nebulizers, UV-C disinfection has also been evaluated [6–8].

In the case of manually applied liquid disinfectants, a standardized dosage is used, which is usually consistently above the tolerance limits for a wide range of pathogens. The occurring errors in the process of manual room disinfection are mostly due to the difficulty in reaching each surface and ensuring the correct contact time during the process in the complex hospital environment [9]. Rarely, there are also consistent dosing errors of the disinfectant [10]. In contrast, automated room disinfection methods generally do not omit relevant surfaces, but can apply very different, not always predictable, doses of the active ingredient to different surfaces in the room, as recently shown for H_2_O_2_ nebulizers [11].

UV-C light is known to be an effective disinfectant against a wide range of microorganisms [12]. But to successfully inactivate pathogens on surfaces, pathogen-dependent germicidal UV-C doses are required. However, different values for germicidal activity against the same pathogens are reported in the current literature [13]. These differences are mainly due to varying conditions of testing. For example, many UV-C doses for the inactivation of pathogens are investigated in air or water, which is different compared to the disinfection of dry surfaces [14, 15]. Furthermore, use of different test organisms and strains of the same pathogen may lead to different results [15]. Also, different conditions of test rooms, laboratory or hospital environment, as well as distances and radiation angle can influence the locally reached dose at different room positions and thus the effectiveness of the disinfection process, especially if shadowing is present [16, 17].

In this context, current guidelines for using automated room disinfection systems recommend that users conduct microbiological verification tests in the respective clinical environment to confirm efficacy [18]. For these efficacy tests, it is important to consider that recent clinical isolates may behave differently compared to laboratory strains, which have been commonly used in the approval process of disinfection procedures or as bio indicators for the control of disinfection processes for decades. This aspect is also considered in the standard procedures for testing chemical disinfectants, as outlined in DIN EN 14885:2019 [19]. The phase 3 testing includes field testing to evaluate the efficacy of new technologies. This involves demonstrating performance under the specific local conditions of the users, using naturally occurring microorganisms [19].

The aim of the following study was to compare the reduction rate of ten different clinical isolates of vancomycin-resistant enterococci (VRE) with *Enterococcus hirae* ATCC 10541, a common test strain used for airborne automatic room disinfection procedures. The comparison was conducted after exposing the samples to UV-C radiation, which the manufacturer specified would achieve a 4 log_10_ reduction on surfaces.

## Methods

To investigate the differences in sensitivity of various clinical isolates of vancomycin-resistant enterococci (VRE) to UV-C radiation, ceramic tiles contaminated with ten different VRE were placed at a distance of 1.0 and 1.5 m from the UVD robot model C (UVD Robots, Odense S, Denmark) and irradiated for 20 s. Since the manufacturer specifies an emittance of minimum 2,500 µW/cm² at a distance of 1 m, the calculated UV-C irradiation doses were 50 mJ/cm² and 22 mJ/cm² at distances of 1.0 and 1.5 m, respectively. The achievement of 50 mJ/cm² was confirmed in individual experiments by the colour change of a UV-C indicator (UVC Dosimeter™, UVD Robots, Odense S, Denmark).

The classical multilocus sequence typing (MLST) sequence types (ST) as well as the core genome MLST (cgMLST) cluster types (CT) were determined from a large strain collection of clinical VRE isolates with available whole genome using the SeqSphere software (Ridom, Münster, Germany) [20]. Ten isolates with the widest possible genomic distance (different ST and/or CT) were selected for further investigations. The occurrence of resistance genes was determined using ResFinder 4.0 [21] and LRE-Finder 1.0 [22, 23].

For the preparation of standardized contaminated surfaces, suspensions of 1.0–5.0 × 10^8^ colony forming units (cfu)/mL were produced for each clinical isolate of VRE. To be representative for realistic protein and dust load of surfaces, 0.3% bovine serum albumin (BSA) solution was used. 20 µL of these suspensions were spread out and dried on ceramic tiles (5 × 5 cm, #3709PN00, Villeroy & Boch, Mettlach, Germany) resulting in microbial burden of approx. 10^6^ cfu/25 cm² (suppl. Fig. S1).

The surfaces were positioned at a distance of 1.0 and 1.5 m to the UVD robot on a one-meter-high table with a black surface in a 6 m³ test room with an anteroom. The white wall behind the table was covered by a black surface, so that the scattered light has minimal influence on the reduction. Within the irradiation process, the UVD robot has a 3-minute warm-up phase. To exclude the effect of irradiation during warm-up from the measurement of the reduction, the warm up was performed in the anteroom. Therefore, the robot was set up to automatically enter the test room after warming up in the anteroom and take up position at a distance of 1 or 1.5 m from the surfaces (suppl. Fig. S2).

For each experiment, four surfaces were positioned at both distances. In each case, two of the surfaces were contaminated with the same VRE isolate. The order of the VRE contaminated surfaces at the distance lines on the table were changed between the experiments to ensure that the collected data were not dependent on the position. In addition, for each VRE isolate two contaminated surfaces were positioned outside the treated room as a control. With this setup, the experiment was performed five times for each of the ten VRE isolates.

During the irradiation, the relative humidity and temperature of the room were measured with LogTag Trex-8 (CiK Solutions GmbH, Karlsruhe, Germany) to ensure that these parameters were constant and had no significant influence on the reduction factors.

Following irradiation, bacteria were recovered from treated and untreated surfaces. To do this, surfaces were wiped off in horizontal, vertical and diagonal directions using flocked nylon swabs (eSwab™ Standard, Copan; Brescia, Italy). Next, the swabs were placed in Aimes Medium and were vortexed for 30 s to elute the recovered bacteria. Afterwards, 100 µL of Aimes medium were spread on Columbia agar with 5% Sheep Blood (bioMérieux, Marcy l’Etoile, France) to perform quantitative culture in double determination (detection limit 5 cfu/25 cm²). Subsequently, the plates were incubated for 18–24 h at 37 °C. Finally, the colony forming units were determined and the mean value of both approaches were calculated.

To calculate the reduction factors, log_10_ of the control were subtracted from the log_10_ of irradiated surfaces. If there was no detection of bacteria, the log_10_ of the control was given as reduction factor, analogue to the specifications in DIN EN 17272:2020 [24].

The differences between the observed reduction factors were statistically analysed by performing one-way ANOVA followed by Tukey post-hoc tests to carry out pairwise comparisons of the groups using R (version 4.0.3) and Rstudio (version 2021.09.1) [25]. Therefore, the R packages *tidyverse, ggpubr, rstatix, psych and car* were activated [26–30]. Preliminary tests to check normality assumption (shapiro_test) and variance homogeneity (residual vs. fits plot) were performed. A Confidence level of 95% was used. For all analyses, *p*-values less than 0.01 were considered statistically significant for the comparison of individual strains, since a significance level of 95% was not considered sufficiently meaningful due to naturally occurring variations in microbial growth.

## Results

**Fig. 1:**
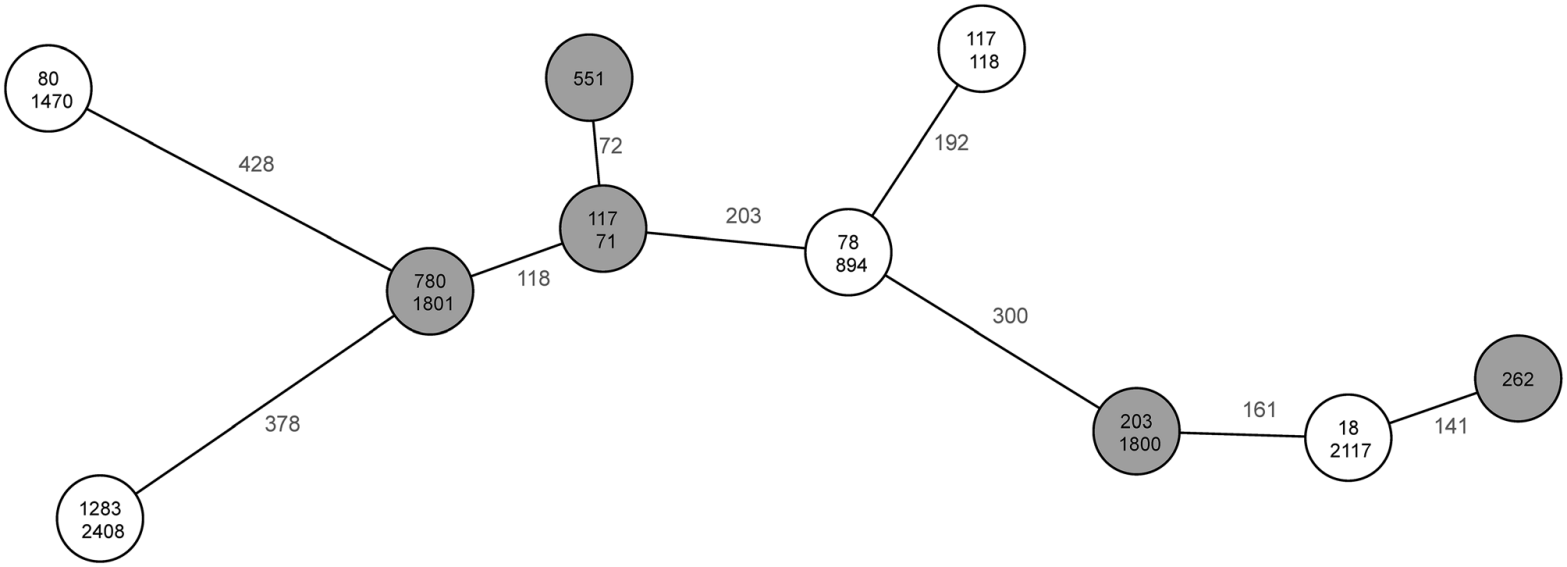
Minimum spanning tree of the core genome sequences of the investigated isolates. Each circle (node) represents one isolate. The number between the nodes indicates the number of allele differences. The upper numbers in each circle indicate the Sequence type (ST). The lower numbers indicate the Cluster type (CT), if applicable. White or gray nodes represent isolates carrying VanA or VanB resistance determinants, respectively

The clinical VRE isolates included in the study showed between 72 and 428 different alleles in cgMLST (Fig. 1). Nine different classical MLST sequence types were represented (ST18, ST80, ST78, ST117, ST203, ST262, ST551, ST780, ST1283), of which the doubly occurring ST117 represented two different cluster types (CT71, CT118) as determined by cgMLST. Five isolates each showed VanA (ST18, ST78, ST80, ST117/CT118, ST1283) or VanB (ST117/CT71, ST203, ST262, ST551, ST780) as resistance mechanism against glycopeptides, respectively. The isolate of ST117/CT71 was reported to be phenotypically resistant against linezolid. Using the LRE-Finder resistance could be genetically confirmed with a wild type/mutant ratio of 57.7/42.3% of the mutation G2576T of the 23 S alleles.

**Fig. 2:**
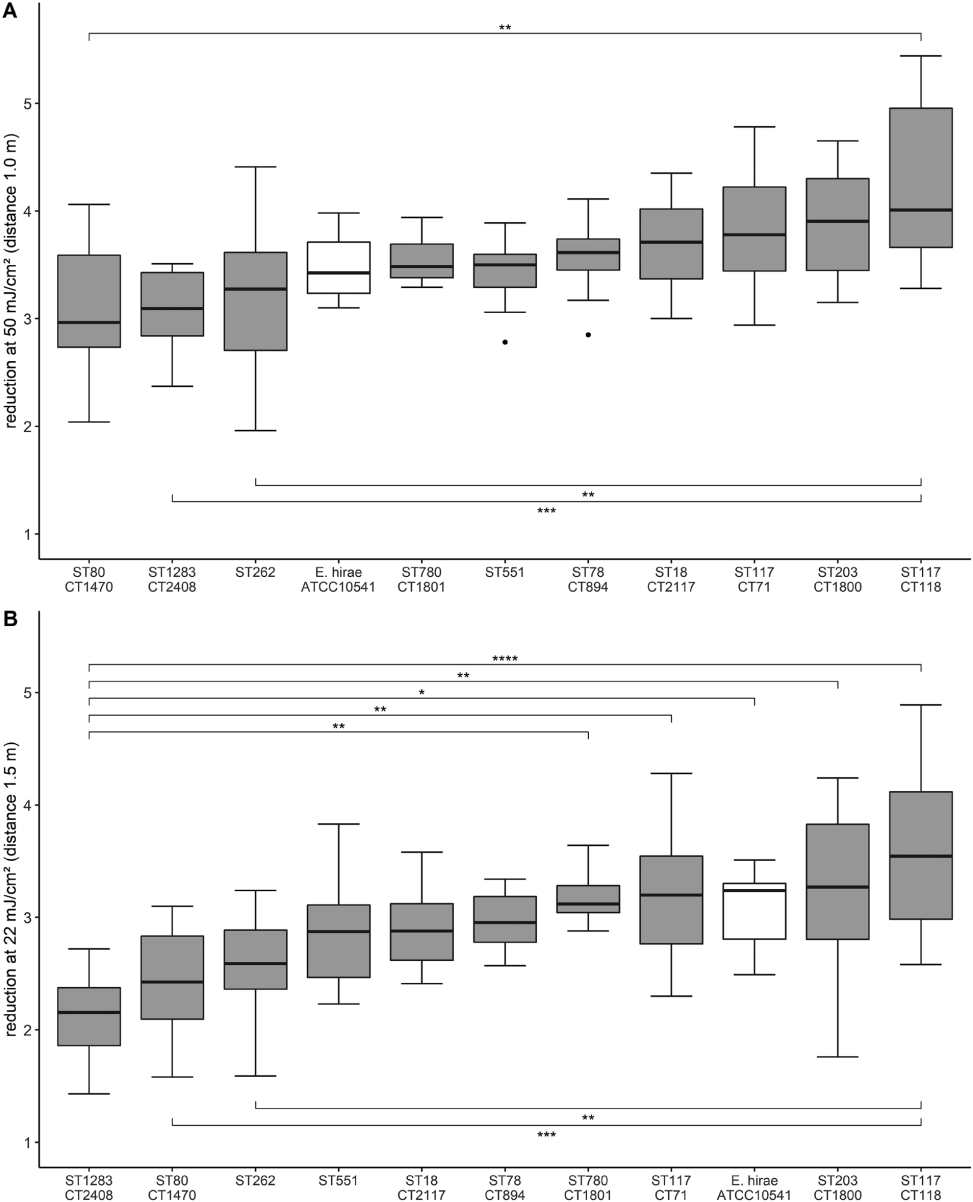
Reduction of different clinical VRE isolates with UV-C doses of 50 **(A)** and 22 mJ/cm² **(B)**. The boxplots represent the reduction factors determined per tested VRE isolate (n = 10) and test organism *E. hirae* ATCC 10541 in 5 independent experiments, comparing microbial load on ceramic tiles before and after UV-C radiation with 50 **(A)** and 22 mJ/cm² **(B)**. Boxplots are in ascending order by median. Statistically significant results (p < 0.01) of pairwise Tukey post-hoc analysis are connected by brackets. Non-significant comparisons are not shown. The p-values are displayed as follows: p < 0.01 = *, p < 0.001 = **, p < 0.0001 = ***, and p < 0.00001 = ****

The high contaminated surfaces used for experiments had an average contamination of 2.3 × 10^6^ cfu/25 cm² (suppl. Fig. S1). Measurement of relative humidity and temperature showed comparative values for all experiments (data not shown). For the UV-C dose of 50 mJ/cm² average reductions between 3.09 and 4.27 log_10_ were achieved (Fig. 2). VRE ST1283 and ST80 were observed to be the most UV-C tolerant of the tested clinical isolates, only reduced by means of 3.09 (median 3.10, SD 0.385) and 3.12 (median 2.96, SD 0.63) log_10_. The greatest reduction was observed for ST117/CT118, reduced by average 4.27 log_10_ (median 4.01, SD 0.82). The common test organism *Enterococcus hirae* ATCC 10541 showed the fourth lowest reduction (mean 3.48 log_10_, median 3.42, SD 0.304).

For the UV-C dose of 22 mJ/cm², the order of the tested clinical isolates slightly changed in terms of the reduction achieved. ST1283 was observed to be the most tolerant isolate with mean reduction of 2.12 log_10_ (median 2.15, SD 0.395) followed by ST80 displaying a mean reduction of 2.41 log_10_ (median 2.42 log_10_, SD 0.518). An average reduction range of 2.12 to 3.61 log_10_ was observed.

One-way ANOVA was conducted to asses if the observed differences in reduction between the ten clinical VRE isolates and the common test organism *E. hirae* ATCC 10541 were statistically significant. The ANOVA was statistically significant for both UV-C doses (50 mJ/cm²: F(10, 99) = 4.702, *p* = 1.72 e-05, generalized eta squared = 0.322; 22 mJ/cm²: F(10, 99) = 6.725, *p* = 6.6 e-08, generalized eta squared = 0.405). Therefore, differences between the tested groups have to be assumed. Subsequent Post-hoc Tukey analyses showed significant differences (*p *< 0.01) in reduction for 50 mJ/cm² between most tolerant isolates ST80 to most susceptible strain ST117/CT118. In addition, reduction of ST117/CT118 is significantly different to ST1283 and ST262. For UV-C dose 22 mJ/cm² statistically significant differences were observed for most tolerant isolate ST1283 to ST780, ST117/CT71, *E. hirae*, ST203 and ST117/CT118. Furthermore, the difference in reduction of the most susceptible strain ST117/CT118 to ST80 and ST262 was statistically significant. The detailed results of Tukey analyses are shown in suppl. Table S3. The difference between the clinical VRE isolates to *E. hirae* ATCC 10541 is statistically significant only compared to ST1283 (-0.961, 95% CI (-1.72 to -0.204), p = 0.0029) when 22 mJ/cm² is applied.

## Discussion

UV-C irradiation of ten highly divers clinical isolates of vancomycin-resistant enterococci (VRE) and a common test organism *Enterococcus hirae* (*E. hirae*) ATCC 10541 showed differences in log_10_ reduction between the tested isolates. For the most tolerant isolate only an average log_10_ reduction of 3.09 and 2.12 was observed at 50 and 22 mJ/cm² UV-C irradiation, respectively, in comparison to the most susceptible one, which showed 4.01 and 3.61 log_10_. The statistical analysis revealed significant differences in the observed reductions between the most tolerant and most susceptible strains. Interestingly, the reduction of clinical isolate ST1283 is significant lower compared to *E. hirae* ATCC 10541, when applying UV-C dose of 22 mJ/cm², indicating that this strain is insufficiently representative of particularly tolerant strains. This is especially important because, in contrast to wipe disinfection with a standardized concentration of the active ingredient, automated room disinfection processes apply a broad dose spectrum of the respective active principle to different surfaces in the room. For the linezolid-resistant VRE ST177/CT71, neither an increased nor a decreased reduction could be observed compared to the other VRE isolates. Therefore, it can be assumed that linezolid resistance has no effect on UV-C tolerance.

Current literature indicates that the UV-C dose required to inactivate bacteria depends on several factors such as type of organism, medium and surface area [17]. For the inactivation of *Enterococcus faecium* on solid surfaces, the manufacturer specifies a dose of 50 mJ/cm², which should be reached with an exposure time of 18.5 s at a distance of 1 m to achieve a reduction of 4 log_10_ [31]. However, this value was derived from laboratory experiments. For the experiments carried out in this study, UV-C doses analogous to the manufacturer’s specifications were used and contaminated surfaces were positioned horizontal but in direct line to the UV-C light source. Under these conditions, the indicated reduction of 4 log_10_ was not achieved in either tested *E. hirae* or clinical isolates of VRE, although horizontally placed UV indicators showed the achievement of the desired dose. This is consistent with the observation made with other methods of room disinfection that, in realistic testing, the dose to be applied must be higher compared to the doses of the disinfection product recommended by the manufacturer and defined in the context of approval studies [11].

Nerandzic *et al.* observed a mean reduction for clinical strains of VRE on benchtop surfaces in a range of approx. 3.2 to 4.4 log_10_ using an UV-C dose of 22 mJ/cm² [15]. Mahida* et al.* also reported a mean reduction of clinical VRE strains seeded on petri dishes greater 4 log_10_ applying 22 mJ/cm² in direct line [32]. Finally, Jelden et al. detected an average reduction range of 3.8 to 4.9 log_10_ for vancomycin-resistant *Enterococcus faecalis* ATCC 51299 on different surface material, using partially much higher UV-C doses in a range of mean 9 to 688 mJ/cm² [17]. In our study, we observed a mean reduction range of 2.12 to 3.61 log_10_ for the ten investigated VRE isolates when using the direct radiation dose of 22 mJ/cm² to disinfect the contaminated ceramic tiles. The observed reductions were up to 1 log_10_ lower than the given literature values.

Nerandzic *et al.* studied three different clinical VRE strains, of which two were VanA and one VanB positive isolates. The obtained results indicated differences in reduction rates of these. While the Van B VRE isolate was reduced just above 3 log_10_, the Van A isolates showed reduction from approx. 3.75 up to just under 4.5 log_10_ [15]. The observed differences between the most tolerant and the most susceptible VRE isolate are in a range of > 1 log_10_. A comparable range was determined in the present study. However, no correlation of more or less susceptibility depending on resistance mechanism against glycopeptides could be observed. The strain with genetically confirmed resistance against glycopeptides displayed no significant differences compared to the laboratory strain *E. hirae* ATCC 10541, whereas a significantly higher reduction was observed for the low radiation dose compared to the most tolerant isolate. This data indicates that mutations in the 23 S alleles do not interfere with the behavior towards UV-C radiation. Both previous data and the results obtained in this study support the recommendation of Beswick *et al.* to conduct tests in the intended clinical environment to verify the efficacy of disinfection methods [18]. This is particularly crucial because manufacturers’ specifications may not provide sufficient guidance to achieve bactericidal efficacy against clinical isolates, which may exhibit higher tolerance than the test organism. Moreover, variations in room conditions, surface materials and shadowing can influence the required UV-C dose for effective disinfection, as evidenced by the previously described differences in reduction at the same UV-C dose. The standard procedure for testing chemical disinfectants, as defined by DIN EN 14885:2019, stipulates a consistent concentration of the active ingredient to guarantee effective disinfection. However, determining a minimum efficacy dose for UV-C is not as straightforward. Consequently, it is advisable to conduct a phase 3 test for automated UV-C disinfection processes to validate their efficacy in routine use across diverse hospital settings. In this regard, more tolerant clinical isolates should be used in the future.

The following limitations of the present study should be noted: Firstly, the experiments were performed with only one standard material. Since several studies show differences depending on surface material (e.g. [17]), further experiments should be performed with other surface materials frequently used in the clinical environment. In addition, it should be noted that there were slight deviations in the disinfection position as part of the robot’s automated travel process. The resulting variation in the source to surface distances could have led to slightly different UV-C doses between the experiments. Furthermore, it has to be taken into account that the experiments were conducted with very high surface contaminations in order to enable calculation of reduction efficacy over a range of several log_10_ reduction. However, realistic contamination of surfaces, even in hospital environments, is much lower [33, 34], so that the disinfection procedure could be effective even if the bactericidal efficacy is not reached. Finally, despite the use of the most diverse VRE isolates as possible, it cannot be excluded that isolates with even higher tolerance to UV-C radiation exist in clinical practice.

## Conclusion

Since manual cleaning and disinfection are frequently inadequate to remove pathogens on surfaces completely, automatic room decontamination with UV-C radiation could be a suitable method to increase efficacy of surface disinfection. However, before using it routinely, efficacy verification is recommended. Testing the efficacy of UV-C radiation for ten different whole-genome-sequenced clinical isolates of VRE as well as ATCC 10541 test organism *Enterococcus hirae*, showed differences in reduction. Use of UV-C radiation according to manufacturer’s instructions did not achieve the proposed reduction. In addition, some clinical isolates of VRE are more stable against UV-C radiation than the common test organism. Therefore, to establish the use of such devices, additional efficacy tests in hospital environments using clinical isolates should be performed.

## Electronic supplementary material

Below is the link to the electronic supplementary material.

Supplementary Material 1

Supplementary Material 2

Supplementary Material 3

## Data Availability

The datasets used and/or analysed during this study are included in this published article and its supplementary information files, further inquiries can be directed to the corresponding author.
