# Supplementary material for: Tolerance of clinical vancomycin-resistant Enterococcus faecium isolates against UV-C light from a mobile source
Source: Antimicrob Resist Infect Control. 2023 Jul 4;12:63. doi: 10.1186/s13756-023-01259-3 (PMC10320914; doi:10.1186/s13756-023-01259-3)
Supplement: Supplementary file 1 — Supplementary Material 1 [file 13756_2023_1259_MOESM1_ESM.docx]

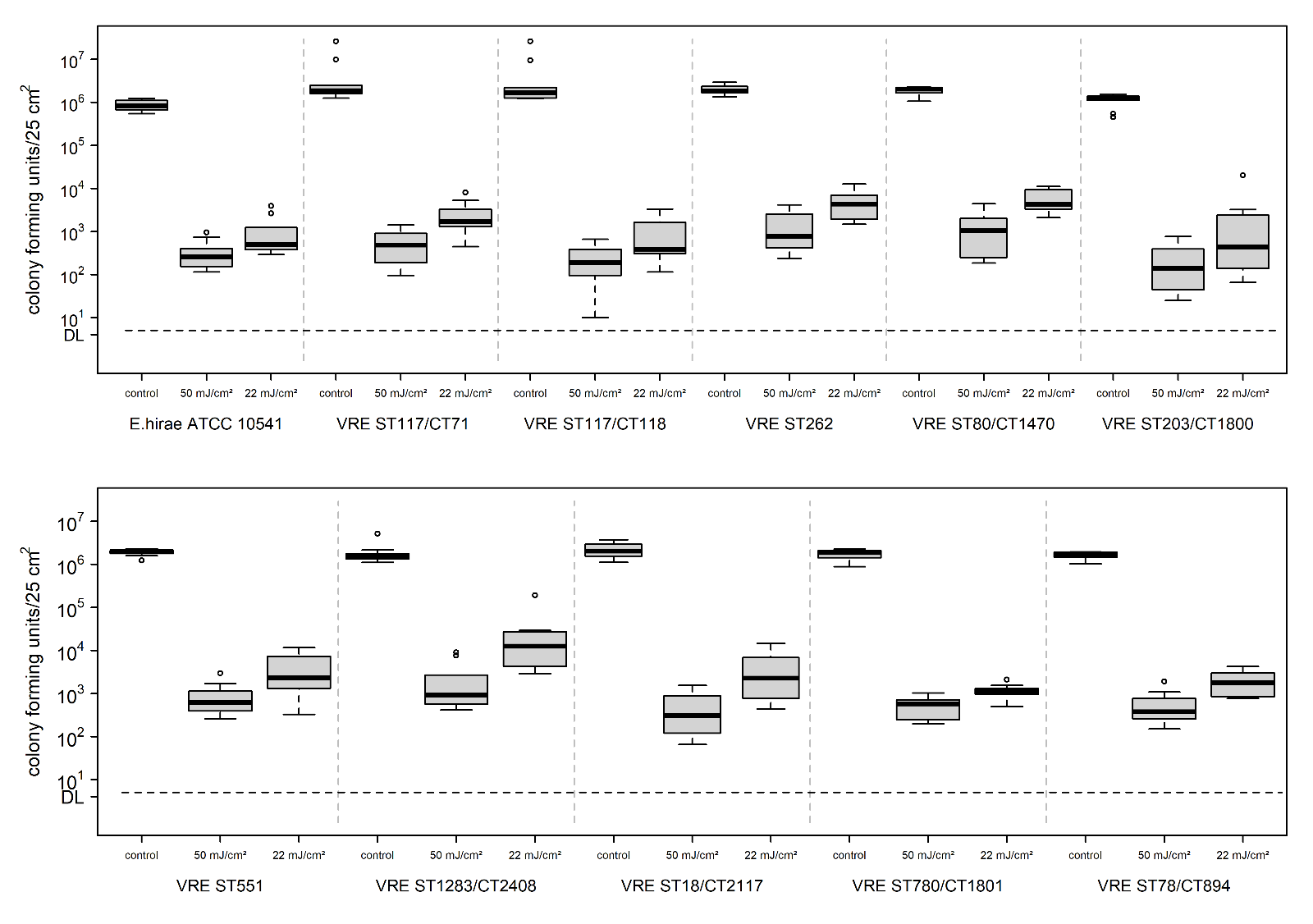


Suppl.Fig.S1 Microbial load of ceramic tiles before and after UV-C radiation with different UV-C doses

The boxplots represent the distribution of total bacterial counts (cfu/25cm²) on ceramic tiles before (control) and after UV-C radiation with 50 mJ/cm² and 22 mJ/cm² (10 values determined in 5 independent experiments). The horizontal dashed line represents the detection limit of the method used (5 cfu/25cm²). The vertical dashed lines separate the results of the *E. hirae* isolate and the clinical vancomycin resistant *E. faecium* (VRE) isolates.
