# Supplementary material for: Tolerance of clinical vancomycin-resistant Enterococcus faecium isolates against UV-C light from a mobile source
Source: Antimicrob Resist Infect Control. 2023 Jul 4;12:63. doi: 10.1186/s13756-023-01259-3 (PMC10320914; doi:10.1186/s13756-023-01259-3)
Supplement: Supplementary file 2 — Supplementary Material 2 [file 13756_2023_1259_MOESM2_ESM.docx]

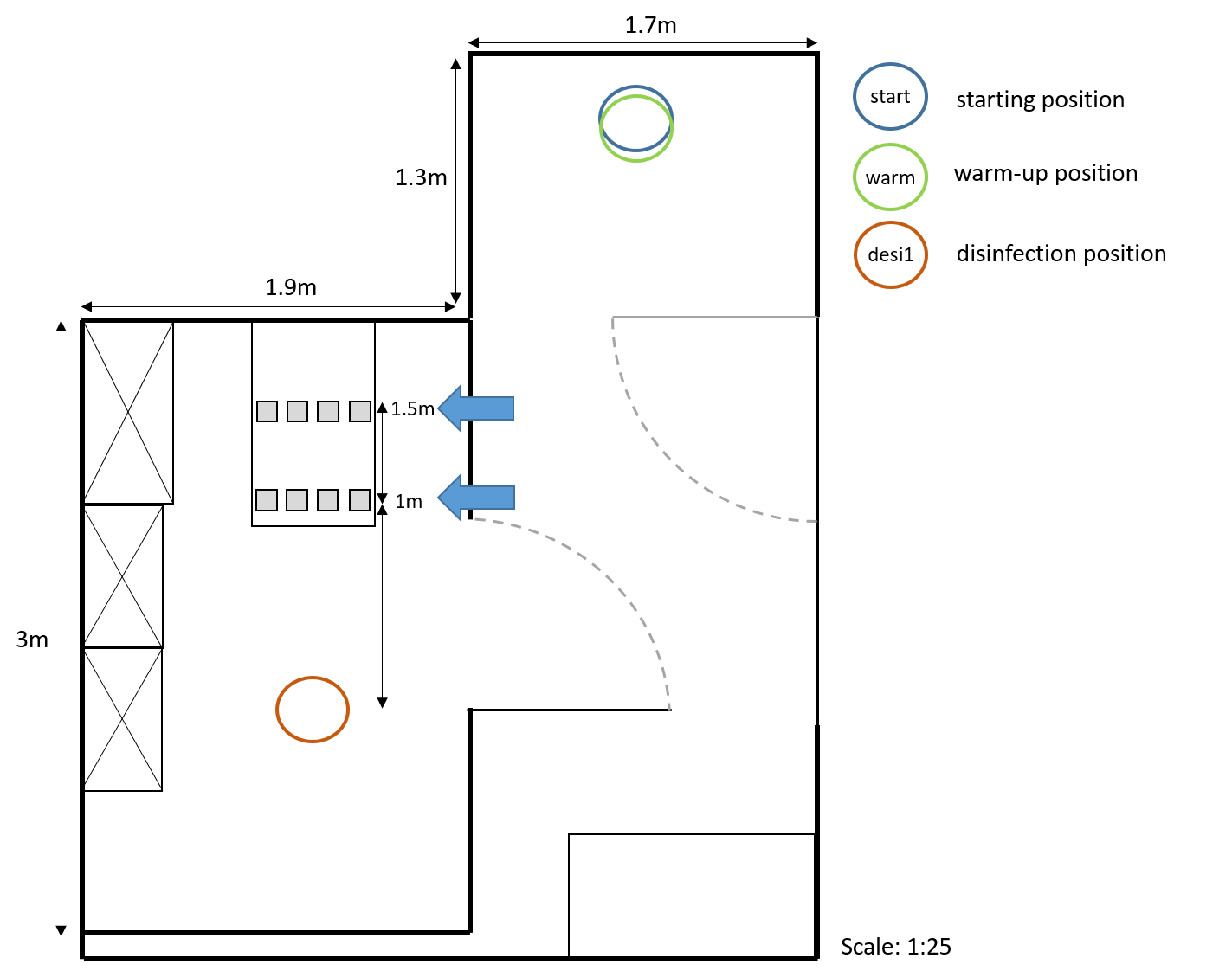


Suppl.Fig.S2: Experimental setup in the test room.

Starting and warm-up position are represented by blue and green circle and positioned in the ante room. After warm-up, the UV-C device moved autonomously to desinfection position represented by the brown circle and located at a distance of 1.0 m and 1.5 m to contaminted ceramic tiles. The gray squares display the contaminated ceramic tiles, which were positioned on a 1 m high black table. The wall behind the table was covered with a black cloth in order to avoid influence of scattered radiation.
