# Supplementary material for: Tolerance of clinical vancomycin-resistant Enterococcus faecium isolates against UV-C light from a mobile source
Source: Antimicrob Resist Infect Control. 2023 Jul 4;12:63. doi: 10.1186/s13756-023-01259-3 (PMC10320914; doi:10.1186/s13756-023-01259-3)
Supplement: Supplementary file 3 — Supplementary Material 3 [file 13756_2023_1259_MOESM3_ESM.docx]

Table 1: Tukey Analyses UV-Dose 50 mJ/cm²

| Strain 1 | Strain 2 | Est-imate | Lower confidence limit | Upper confidence limit | Adjusted  p-value | Adjusted p-value significance |
| --- | --- | --- | --- | --- | --- | --- |
| E. hirae | ST117a | 0,331 | -0,43449546 | 1,09649546 | 0,939 | ns |
| E. hirae | ST117b | 0,791 | 0,02550454 | 1,55649546 | 0,0366 | ns |
| E. hirae | ST1283 | -0,394 | -1,15949546 | 0,37149546 | 0,834 | ns |
| E. hirae | ST18 | 0,22 | -0,54549546 | 0,98549546 | 0,997 | ns |
| E. hirae | ST203 | 0,398 | -0,36749546 | 1,16349546 | 0,825 | ns |
| E. hirae | ST262 | -0,284 | -1,04949546 | 0,48149546 | 0,978 | ns |
| E. hirae | ST551 | -0,052 | -0,81749546 | 0,71349546 | 1 | ns |
| E. hirae | ST78 | 0,08 | -0,68549546 | 0,84549546 | 1 | ns |
| E. hirae | ST780 | 0,064 | -0,70149546 | 0,82949546 | 1 | ns |
| E. hirae | ST80 | -0,366 | -1,13149546 | 0,39949546 | 0,889 | ns |
| ST117a | ST117b | 0,46 | -0,30549546 | 1,22549546 | 0,663 | ns |
| ST117a | ST1283 | -0,725 | -1,49049546 | 0,04049546 | 0,08 | ns |
| ST117a | ST18 | -0,111 | -0,87649546 | 0,65449546 | 1 | ns |
| ST117a | ST203 | 0,067 | -0,69849546 | 0,83249546 | 1 | ns |
| ST117a | ST262 | -0,615 | -1,38049546 | 0,15049546 | 0,24 | ns |
| ST117a | ST551 | -0,383 | -1,14849546 | 0,38249546 | 0,857 | ns |
| ST117a | ST78 | -0,251 | -1,01649546 | 0,51449546 | 0,991 | ns |
| ST117a | ST780 | -0,267 | -1,03249546 | 0,49849546 | 0,986 | ns |
| ST117a | ST80 | -0,697 | -1,46249546 | 0,06849546 | 0,109 | ns |
| ST117b | ST1283 | -1,185 | -1,95049546 | -0,41950454 | 8,43E-05 | *** |
| ST117b | ST18 | -0,571 | -1,33649546 | 0,19449546 | 0,342 | ns |
| ST117b | ST203 | -0,393 | -1,15849546 | 0,37249546 | 0,836 | ns |
| ST117b | ST262 | -1,075 | -1,84049546 | -0,30950454 | 0,000558 | ** |
| ST117b | ST551 | -0,843 | -1,60849546 | -0,07750454 | 0,0186 | ns |
| ST117b | ST78 | -0,711 | -1,47649546 | 0,05449546 | 0,0934 | ns |
| ST117b | ST780 | -0,727 | -1,49249546 | 0,03849546 | 0,0782 | ns |
| ST117b | ST80 | -1,157 | -1,92249546 | -0,39150454 | 0,000138 | ** |
| ST1283 | ST18 | 0,614 | -0,15149546 | 1,37949546 | 0,242 | ns |
| ST1283 | ST203 | 0,792 | 0,02650454 | 1,55749546 | 0,0361 | ns |
| ST1283 | ST262 | 0,11 | -0,65549546 | 0,87549546 | 1 | ns |
| ST1283 | ST551 | 0,342 | -0,42349546 | 1,10749546 | 0,925 | ns |
| ST1283 | ST78 | 0,474 | -0,29149546 | 1,23949546 | 0,622 | ns |
| ST1283 | ST780 | 0,458 | -0,30749546 | 1,22349546 | 0,669 | ns |
| ST1283 | ST80 | 0,028 | -0,73749546 | 0,79349546 | 1 | ns |
| ST18 | ST203 | 0,178 | -0,58749546 | 0,94349546 | 1 | ns |
| ST18 | ST262 | -0,504 | -1,26949546 | 0,26149546 | 0,532 | ns |
| ST18 | ST551 | -0,272 | -1,03749546 | 0,49349546 | 0,984 | ns |
| ST18 | ST78 | -0,14 | -0,90549546 | 0,62549546 | 1 | ns |
| ST18 | ST780 | -0,156 | -0,92149546 | 0,60949546 | 1 | ns |
| ST18 | ST80 | -0,586 | -1,35149546 | 0,17949546 | 0,305 | ns |
| ST203 | ST262 | -0,682 | -1,44749546 | 0,08349546 | 0,127 | ns |
| ST203 | ST551 | -0,45 | -1,21549546 | 0,31549546 | 0,692 | ns |
| ST203 | ST78 | -0,318 | -1,08349546 | 0,44749546 | 0,953 | ns |
| ST203 | ST780 | -0,334 | -1,09949546 | 0,43149546 | 0,936 | ns |
| ST203 | ST80 | -0,764 | -1,52949546 | 0,00149546 | 0,0509 | ns |
| ST262 | ST551 | 0,232 | -0,53349546 | 0,99749546 | 0,995 | ns |
| ST262 | ST78 | 0,364 | -0,40149546 | 1,12949546 | 0,892 | ns |
| ST262 | ST780 | 0,348 | -0,41749546 | 1,11349546 | 0,917 | ns |
| ST262 | ST80 | -0,082 | -0,84749546 | 0,68349546 | 1 | ns |
| ST551 | ST78 | 0,132 | -0,63349546 | 0,89749546 | 1 | ns |
| ST551 | ST780 | 0,116 | -0,64949546 | 0,88149546 | 1 | ns |
| ST551 | ST80 | -0,314 | -1,07949546 | 0,45149546 | 0,957 | ns |
| ST78 | ST780 | -0,016 | -0,78149546 | 0,74949546 | 1 | ns |
| ST78 | ST80 | -0,446 | -1,21149546 | 0,31949546 | 0,703 | ns |
| ST780 | ST80 | -0,43 | -1,19549546 | 0,33549546 | 0,747 | ns |

Table 2: Tukey Analyses UV-Dose 22 mJ/cm²

| Strain 1 | Strain 2 | Est-imate | Lower confidence limit | Upper confidence limit | Adjusted  p-value | Adjusted p-value significance |
| --- | --- | --- | --- | --- | --- | --- |
| E. hirae | ST117a | 0,074 | -0,68280886 | 0,83080886 | 1 | ns |
| E. hirae | ST117b | 0,526 | -0,23080886 | 1,28280886 | 0,449 | ns |
| E. hirae | ST1283 | -0,961 | -1,71780886 | -0,20419114 | 0,0029 | * |
| E. hirae | ST18 | -0,155 | -0,91180886 | 0,60180886 | 1 | ns |
| E. hirae | ST203 | 0,166 | -0,59080886 | 0,92280886 | 1 | ns |
| E. hirae | ST262 | -0,541 | -1,29780886 | 0,21580886 | 0,406 | ns |
| E. hirae | ST551 | -0,224 | -0,98080886 | 0,53280886 | 0,996 | ns |
| E. hirae | ST78 | -0,11 | -0,86680886 | 0,64680886 | 1 | ns |
| E. hirae | ST780 | 0,095 | -0,66180886 | 0,85180886 | 1 | ns |
| E. hirae | ST80 | -0,668 | -1,42480886 | 0,08880886 | 0,135 | ns |
| ST117a | ST117b | 0,452 | -0,30480886 | 1,20880886 | 0,671 | ns |
| ST117a | ST1283 | -1,035 | -1,79180886 | -0,27819114 | 0,000886 | ** |
| ST117a | ST18 | -0,229 | -0,98580886 | 0,52780886 | 0,995 | ns |
| ST117a | ST203 | 0,092 | -0,66480886 | 0,84880886 | 1 | ns |
| ST117a | ST262 | -0,615 | -1,37180886 | 0,14180886 | 0,225 | ns |
| ST117a | ST551 | -0,298 | -1,05480886 | 0,45880886 | 0,967 | ns |
| ST117a | ST78 | -0,184 | -0,94080886 | 0,57280886 | 0,999 | ns |
| ST117a | ST780 | 0,021 | -0,73580886 | 0,77780886 | 1 | ns |
| ST117a | ST80 | -0,742 | -1,49880886 | 0,01480886 | 0,0597 | ns |
| ST117b | ST1283 | -1,487 | -2,24380886 | -0,73019114 | 2,00E-07 | *** |
| ST117b | ST18 | -0,681 | -1,43780886 | 0,07580886 | 0,118 | ns |
| ST117b | ST203 | -0,36 | -1,11680886 | 0,39680886 | 0,892 | ns |
| ST117b | ST262 | -1,067 | -1,82380886 | -0,31019114 | 0,00052 | ** |
| ST117b | ST551 | -0,75 | -1,50680886 | 0,00680886 | 0,0543 | ns |
| ST117b | ST78 | -0,636 | -1,39280886 | 0,12080886 | 0,186 | ns |
| ST117b | ST780 | -0,431 | -1,18780886 | 0,32580886 | 0,731 | ns |
| ST117b | ST80 | -1,194 | -1,95080886 | -0,43719114 | 5,62E-05 | *** |
| ST1283 | ST18 | 0,806 | 0,04919114 | 1,56280886 | 0,0269 | ns |
| ST1283 | ST203 | 1,127 | 0,37019114 | 1,88380886 | 0,000185 | ** |
| ST1283 | ST262 | 0,42 | -0,33680886 | 1,17680886 | 0,76 | ns |
| ST1283 | ST551 | 0,737 | -0,01980886 | 1,49380886 | 0,0634 | ns |
| ST1283 | ST78 | 0,851 | 0,09419114 | 1,60780886 | 0,0146 | ns |
| ST1283 | ST780 | 1,056 | 0,29919114 | 1,81280886 | 0,000625 | ** |
| ST1283 | ST80 | 0,293 | -0,46380886 | 1,04980886 | 0,971 | ns |
| ST18 | ST203 | 0,321 | -0,43580886 | 1,07780886 | 0,946 | ns |
| ST18 | ST262 | -0,386 | -1,14280886 | 0,37080886 | 0,842 | ns |
| ST18 | ST551 | -0,069 | -0,82580886 | 0,68780886 | 1 | ns |
| ST18 | ST78 | 0,045 | -0,71180886 | 0,80180886 | 1 | ns |
| ST18 | ST780 | 0,25 | -0,50680886 | 1,00680886 | 0,991 | ns |
| ST18 | ST80 | -0,513 | -1,26980886 | 0,24380886 | 0,487 | ns |
| ST203 | ST262 | -0,707 | -1,46380886 | 0,04980886 | 0,0893 | ns |
| ST203 | ST551 | -0,39 | -1,14680886 | 0,36680886 | 0,833 | ns |
| ST203 | ST78 | -0,276 | -1,03280886 | 0,48080886 | 0,981 | ns |
| ST203 | ST780 | -0,071 | -0,82780886 | 0,68580886 | 1 | ns |
| ST203 | ST80 | -0,834 | -1,59080886 | -0,07719114 | 0,0185 | ns |
| ST262 | ST551 | 0,317 | -0,43980886 | 1,07380886 | 0,95 | ns |
| ST262 | ST78 | 0,431 | -0,32580886 | 1,18780886 | 0,731 | ns |
| ST262 | ST780 | 0,636 | -0,12080886 | 1,39280886 | 0,186 | ns |
| ST262 | ST80 | -0,127 | -0,88380886 | 0,62980886 | 1 | ns |
| ST551 | ST78 | 0,114 | -0,64280886 | 0,87080886 | 1 | ns |
| ST551 | ST780 | 0,319 | -0,43780886 | 1,07580886 | 0,948 | ns |
| ST551 | ST80 | -0,444 | -1,20080886 | 0,31280886 | 0,694 | ns |
| ST78 | ST780 | 0,205 | -0,55180886 | 0,96180886 | 0,998 | ns |
| ST78 | ST80 | -0,558 | -1,31480886 | 0,19880886 | 0,359 | ns |
| ST780 | ST80 | -0,763 | -1,51980886 | -0,00619114 | 0,0464 | ns |
